# Androgen Receptor Expression and T-Lymphocyte Infiltration as Prognostic Indicators in Triple-Negative Breast Cancer: A Retrospective Study

**DOI:** 10.3390/biomedicines14061325

**Published:** 2026-06-11

**Authors:** Olga Milbrandt, Mateusz Wichtowski, Justyna Marcinkowska, Monika Krzyżaniak, Kamil Pytlak, Rodryg Ramlau, Paweł Kurzawa

**Affiliations:** 1 Department of Chemotherapy, Institute of Oncology, Poznan University of Medical Sciences, 60-569 Poznan, Poland; olga.milbrandt@usk.poznan.pl (O.M.); rodryg.ramlau@usk.poznan.pl (R.R.); 2 Department of Surgical Oncology, Institute of Oncology, Poznan University of Medical Sciences, 60-569 Poznan, Poland; 3 Department of Computer Science and Statistics, Poznan University of Medical Sciences, 61-701 Poznan, Poland; justyna.marcinkowska@ump.edu.pl; 4 Department of Clinical Pathology and Immunology, Poznan University of Medical Sciences, 61-701 Poznan, Poland; monika.krzyzaniak@usk.poznan.pl (M.K.); pawel.kurzawa@usk.poznan.pl (P.K.); 5 Independent Researcher, 50-060 Wroclaw, Poland; 6 Department of Oncological Pathology, University Clinical Hospital, 60-569 Poznan, Poland

**Keywords:** triple-negative breast cancer (TNBC), androgen receptor (AR), tumor-infiltrating lymphocytes (TILs), CD4^+^, CD8^+^, prognosis

## Abstract

**Background:** Triple-negative breast cancer (TNBC) is a biologically heterogeneous disease. Clinically accessible biomarkers remain limited. **Objective:** To evaluate androgen receptor (AR) expression and immune response—stromal tumor-infiltrating lymphocytes (sTILs), CD4^+^, CD8^+^, CD4/CD8 ratio—to explore their clinicopathological associations and relationships with 3-year overall survival (OS) in TNBC. **Methods:** We retrospectively analyzed data from 86 treatment-naïve women with TNBC who were treated between 2012 and 2019 at a single academic center. AR and CD4/CD8 were assessed immunohistochemically; sTILs were scored on H&E following The International TIL Working Group recommendations. Survival analyses focused on 3-year OS, with follow-up truncated at 36 months and multivariable Cox regression restricted to non-metastatic disease (stage I–III). **Results:** High AR expression (≥10%) occurred in 23% of tumors and was associated with lower CD8^+^ infiltration and lower tumor grade. Across adjusted models, we did not demonstrate statistically significant and independent associations between AR or immune markers and 3-year OS; however, inference is limited by the low number of events (10 deaths). **Conclusions:** AR status was associated with the immune response, particularly with reduced CD8^+^ infiltration in AR-high tumors, supporting the concept of biologically distinct AR–immune phenotypes. The absence of statistically significant survival associations in adjusted analyses should be interpreted cautiously given the limited event counts, and larger prospective cohorts are needed to validate prognostic and potential therapeutic implications.

## 1. Introduction

Triple-negative breast cancer (TNBC) is a biologically aggressive subtype of breast cancer characterized by the absence of estrogen receptor (ER), progesterone receptor (PR), and human epidermal growth factor receptor 2 (HER2) expression. It accounts for approximately 15–20% of all newly diagnosed breast cancers and is associated with a high histological grade, early recurrence, and poor prognosis. While TNBC lacks a defined molecular target, chemotherapy remains the standard of care across early and advanced stages of the disease [1,2,3].

Clinically, TNBC is a heterogeneous disease. Subgroups of patients differ in clinical course, response to treatment, and overall outcomes. Several molecular classifications have been proposed to address this heterogeneity. Notably, Lehmann et al. and Jiang et al. described transcriptome-based TNBC subtypes, including the luminal androgen receptor (LAR), immunomodulatory (IM), basal-like (BL1/BL2), mesenchymal (MES), and basal-like immunosuppressive (BLIS) types [4,5,6]. However, these molecular classifications have limited applicability in routine clinical practice due to the need for complex transcriptomic RNA-based profiling.

The tumor immune microenvironment has emerged as a key factor in TNBC prognosis. Tumor-infiltrating lymphocytes (TILs), particularly those located in the tumor stroma, are commonly found in TNBC and are associated with both response to chemotherapy and improved survival [7,8]. The International Immuno-Oncology Working Group has published standardized guidelines for assessing stromal TILs, and recent consensus panels have endorsed TILs as a prognostic marker in early-stage TNBC [9,10,11].

Another potential biomarker of interest in TNBC is androgen receptor (AR) expression. AR is found in a subset of TNBC cases, particularly in the LAR subtype. Studies on the prognostic role of AR have yielded conflicting results, with reports suggesting favorable, unfavorable, or context-dependent associations depending on cohort composition and methodology [5,12,13]. Furthermore, anti-androgen therapies are being explored as targeted options for AR-positive TNBC, making AR assessment potentially relevant in clinical practice [14,15].

Despite growing evidence, there remains a need for accessible biomarkers that can be implemented in routine pathology laboratories to stratify patients by prognosis and potential therapeutic benefit. Despite accumulating evidence supporting stromal TILs as prognostic and predictive biomarkers in TNBC, translating immune metrics into routine risk stratification remains challenging due to heterogeneity in patient populations, endpoints, and threshold definitions. International recommendations emphasize standardized assessment of stromal TILs and encourage continuous reporting. Nevertheless, categorical cut-offs continue to vary across studies and clinical contexts [8,9,11,16].

Similarly, the clinical significance of AR expression in TNBC remains controversial, partly because AR measurement methods and positivity thresholds are not harmonized. Recent work in a large population-based cohort using automated digital image analysis identified a 10% AR threshold as prognostically informative for distant metastasis-free survival. It also highlighted a strong interaction between lymph node status and AR expression, consistent with context-dependent (“dual”) roles of AR in TNBC biology and outcomes [17].

Importantly, emerging clinical–pathological frameworks support the evaluation of AR expression within the immune microenvironment rather than as an isolated marker. For example, AR/TIL phenotyping in contemporary TNBC cohorts has been used to identify subgroups with distinct treatment response patterns, including neoadjuvant chemotherapy resistance in AR-positive/TIL-low tumors [18]. In addition, molecularly informed classifications link AR-defined phenotypes with immune-depleted groups and distinct genomic features, reinforcing the biological plausibility of AR–immune interaction in TNBC [19].

Therefore, the gap we address is the limited availability of pathology-based data integrating AR with immune response metrics (sTILs, CD4^+^, CD8^+^, CD4/CD8 ratio) in a treatment-naïve Central/Eastern European cohort using standardized immune scoring. Our contribution is to (i) characterize the relationship between AR expression and immune infiltration (including CD8^+^) and (ii) explore their association with short-term overall survival while explicitly acknowledging limitations of the number of patients.

### Aim of the Study

This study sought to comprehensively evaluate the clinical significance of two emerging biomarkers in triple-negative breast cancer (TNBC)—stromal tumor-infiltrating lymphocytes (sTILs) and androgen receptor (AR) expression. Our primary focus was to examine the relationship between AR expression and sTIL infiltration patterns and to assess their combined prognostic value in TNBC patients. We further aimed to explore how these biomarkers correlate with standard clinicopathological parameters and influence 3-year overall survival outcomes. By integrating immune (sTILs) and hormonal (AR) expression, this study intended to identify clinically relevant TNBC subgroups with distinct biological characteristics and survival patterns, potentially paving the way for more personalized treatment approaches for this aggressive breast cancer subtype.

## 2. Materials and Methods

This retrospective study included 86 female patients diagnosed with triple-negative breast cancer (TNBC) and treated at the Department of Chemotherapy, Poznan University of Medical Sciences between 2012 and 2019. From a total cohort of 6000 breast cancer patients, 720 were identified as TNBC based on pathology reports, and 116 met the initial inclusion criteria. Due to insufficient tumor tissue for immunohistochemistry or lack of consent, the final study population consisted of 86 cases. Inclusion criteria are described in Table 1.

All procedures were approved by the Bioethics Committee of the Poznan University of Medical Sciences (resolution no. 49/17). The study was conducted in accordance with the Declaration of Helsinki.

### 2.1. Evaluation of Stromal Tumor-Infiltrating Lymphocytes (TILs)

The FFPE tumor tissue was sectioned at 4 µm and stained with Mayer’s hematoxylin and eosin (H&E) using an automated multistainer (Leica ST5020, Nussloch, Germany) with integrated coverslipper (Leica CV5030). Stromal TILs were assessed following the recommendations of the International TIL Working Group [9].

TILs were evaluated as the percentage of stromal area infiltrated by mononuclear inflammatory cells, excluding areas of necrosis, artifacts, ductal carcinoma in situ (DCIS), or peritumoral tissue. Quantification was performed at 200× and 400× magnification by an experienced pathologist blinded to clinical outcomes. Only stromal TILs were included. Formal interobserver reproducibility assessment was not performed, given known interobserver variability in manual TIL scoring, which is acknowledged as a limitation [20].

All immunohistochemical (IHC) analyses were performed on the Dako Autostainer Link 48 (Glostrup, Denmark) using the EnVision™ FLEX High pH detection system (Santa Clara, CA, USA). Antigen retrieval was conducted with the Dako PT Link system at 97 °C for 20 min using Target Retrieval Solution (High pH). All antibodies and reagents used in the study are presented in Table 2.

### 2.2. Scoring Criteria

AR expression was assessed as the percentage of positively stained tumor cell nuclei. AR was evaluated both as a continuous variable and dichotomized using the clinically relevant threshold of ≥10% to define high AR expression. This threshold is widely used in TNBC research and is supported by recent evidence from larger cohorts using objective quantification, including digital image analysis, in which ≥10% AR was identified as a prognostically informative cut-off in specific clinical contexts [17].

The Ki-67 proliferation index was quantified as a continuous percentage. TILs were assessed on H&E-stained sections following the International TIL Working Group recommendations and reported as continuous stromal percentages, with predefined categorical cut-offs used for subgroup analyses (e.g., ≤10% vs. >10%, ≤60% vs. >60%) [9].

The 10% threshold was used to distinguish low from higher immune infiltration in descriptive analyses, whereas the 60% threshold was used to identify a lymphocyte-predominant subgroup, consistent with commonly used immune stratifications in TNBC. We acknowledge that categorical TIL thresholds vary across studies and clinical endpoints (e.g., ≥40% has been used in neoadjuvant pCR-focused cohorts), and thus categorical analyses are intended to be descriptive alongside continuous reporting [8,9,16].

Continuous variables (AR%, TIL%, Ki-67%) were summarized using means with standard deviations, medians with interquartile ranges and full ranges. Group comparisons for continuous variables were performed using the permutation-based Welch *t*-test, which provides robust inference under heteroscedasticity and non-normality while maintaining valid type I error rates.

Additionally, the CD4/CD8 ratio was calculated for each tumor sample. Based on published data [21], a cut-off value of >1.2 was used to define a high CD4/CD8 ratio. As tumor-based CD4/CD8 thresholds are not standardized and have not been widely validated specifically for TNBC, this dichotomization should be considered exploratory. Accordingly, results involving the CD4/CD8 cut-off are interpreted cautiously and primarily as hypothesis-generating. This parameter was analyzed descriptively and in relation to 3-year overall survival using Kaplan–Meier and Cox regression models.

Associations between continuous variables were evaluated using Spearman’s rank correlation. Categorical variables—including AR status, TIL categories, tumor grade, menopausal state, and ECOG status—were compared using Fisher’s exact test, and effect sizes were quantified using Cramer’s V.

Overall survival (OS) was defined as the interval from diagnosis to death from any cause. For analyses of 3-year OS, survival times beyond 36 months were right-censored at 36 months, and deaths occurring after this time were treated as censored observations. Survival curves were estimated using the Kaplan–Meier method, and differences between groups were tested using the log-rank test.

Given the low number of deaths within the 36-month window (10 events in the full cohort), survival analyses—particularly multivariable Cox regression—are underpowered and may yield wide confidence intervals. Therefore, non-significant associations should not be interpreted as evidence of absence of effect, and estimates are presented as exploratory and hypothesis-generating.

Multivariable prognostic modeling was performed using Cox proportional hazards regression, restricted to patients with non-metastatic (stage I–III) disease. Clinically relevant covariates included age at diagnosis, ECOG performance status (≥1 vs. 0), anatomical stage (advanced vs. early), Ki-67 index, AR expression (%), and TILs (%). Model results are reported as hazard ratios (HR) with 95% confidence intervals (CI). Proportional hazards assumptions were checked using Schoenfeld residuals.

All statistical analyses were performed using Statistica v13.1 (StatSoft, license JPZ711B306627AR-V) and R (v4.4.2).

## 3. Results

The study cohort included 86 female patients diagnosed with triple-negative breast cancer (TNBC). The median age at diagnosis was 57 years (IQR 45–66), with a mean of 56.0 ± 14.0 years (range 28–87). Postmenopausal women predominated (64%), while 36% were perimenopausal at the time of diagnosis. The mean body mass index (BMI) was 26.7 ± 5.8 kg/m^2^, and the median BMI was 24.9 kg/m^2^ (IQR 23.2–30.8). Nearly half of the patients had a normal BMI (47%), while 23% were overweight and 26% were obese. Four patients (4.7%) were underweight. Most participants had good performance status (ECOG 0–1: 96%), and only three (3.5%) had ECOG 2. BRCA mutation testing was available for 19 patients, of whom 15 (79%) were BRCA1/2-positive. The median Ki-67 proliferation index was 0.60 (IQR 0.35–0.80), indicating generally high proliferative activity. Regarding tumor extent, T2 lesions were most common (41%), followed by T1 (36%). Nodal involvement was absent in the majority of patients (N0: 58%), while 24% had N1 disease. Distant metastases (M1) were present in 12% of patients at diagnosis. According to the AJCC anatomic stage grouping, most cases were classified as stage I–II (69%), while 12% presented with stage IV disease (Table 3).

**Figure 1:**
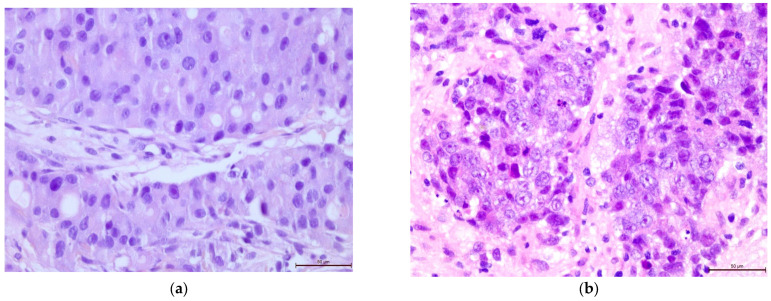

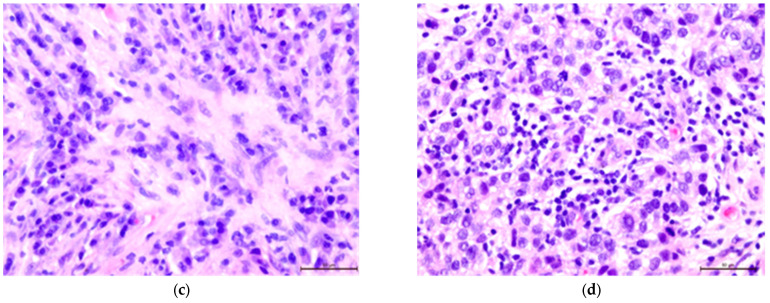
Invasive breast cancer with TILs at 5% (**a**), 20% (**b**), 70% (**c**), and 90% (**d**) infiltration in the tumor stroma, H&E staining, area 400× mag.

Stromal TILs were present in all tumors (100%), with a median infiltration of 20% (range: 1–90%). High TIL infiltration (≥60%) was observed in 15% of tumors, whereas low infiltration (≤10%) was seen in 33% of cases (Figure 1).

**Figure 2:**
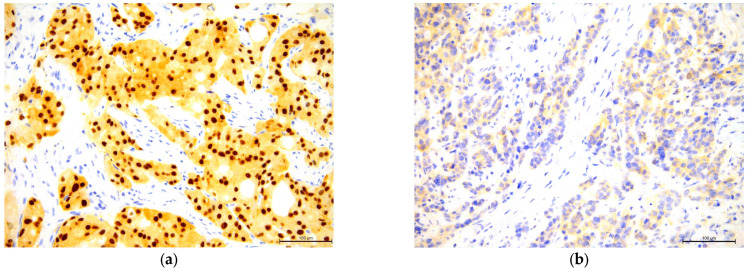
Invasive breast cancer showing the lack (AR = 0) (**a**) and 90% (**b**) nuclear expression of the AR using anti-AR in the area of 200× mag.

AR expression was detected in 28% of tumors, with a median of 0% (range: 0–95%). Low AR expression (<10%) was observed in 77% of tumors, whereas high expression (≥10%) was observed in 23% of cases (Table 4, Figure 2).

A non-significant negative correlation was found between AR expression and total TILs (Spearman’s ρ = −0.16, *p* = 0.143). Mean stromal TIL infiltration was slightly lower in AR-high tumors compared with AR-low tumors (0.22 vs. 0.30), but this difference did not reach statistical significance in the permutation Welch test (*p* = 0.16). The estimated mean difference indicated a small, non-significant reduction of 0.08 (95% CI: −0.03 to 0.20).

Similarly, CD4^+^ T-cell infiltration showed no significant association with AR expression level (*p* = 0.39). Mean CD4^+^ infiltration was 0.16 in the AR-high group and 0.19 in the AR-low group, with a mean difference of 0.04 (95% CI: −0.05 to 0.12).

**Figure 3:**
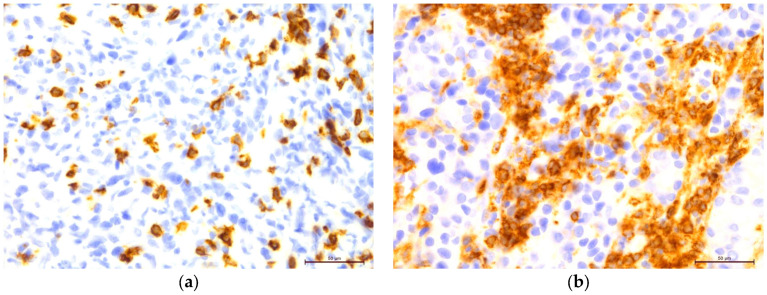
Invasive breast cancer with 70% T-lymphocyte infiltration in the tumor stroma using anti-CD4 (**a**) and anti-CD8 (**b**) in the area of 400× mag.

In contrast, CD8^+^ infiltration differed significantly between groups (*p* = 0.020). Tumors with high AR expression exhibited lower CD8^+^ levels (mean 0.06 vs. 0.10 in the AR-low group). The mean difference of 0.04 (95% CI: 0.01 to 0.08) indicates reduced cytotoxic T-cell infiltration in AR-high tumors. The CD4/CD8 ratio tended to be higher in AR-high tumors, although the difference did not reach statistical significance (*p* = 0.079) (Table 5, Figure 3).

Low stromal TIL infiltration (≤10%) was observed in 28 patients (33%), whereas 58 patients (67%) had TILs >10%. TIL infiltration level was significantly associated with tumor grade (*p* = 0.039). Tumors with low TILs showed a higher prevalence of high-grade disease (G3), accounting for 66% of cases in the ≤10% TIL group compared with 39% in the >10% group. Conversely, intermediate-grade tumors (G2) were more frequent among patients with higher TIL infiltration (57% vs. 33%). The effect size was small-to-moderate (Cramer’s V = 0.249). No association was found between TIL infiltration and high Ki-67 proliferation status defined by the ≥20% cut-off (93% vs. 93%; Fisher’s exact *p* > 0.99) (Table 6).

High AR expression (≥10%) was identified in 20 patients (23%). AR status was strongly associated with tumor grade (*p* < 0.001). Among patients with high AR expression, 85% had G2 tumors, whereas only 5% had G3 carcinoma. In contrast, G3 tumors predominated in the AR-low group (73%), with no G1 tumors observed. This association demonstrated a large effect size (Cramer’s V = 0.605), indicating a robust relationship between AR expression and tumor differentiation. No significant association was observed between AR expression and high Ki-67 proliferation status (≥20%) (85% vs. 95%; *p* = 0.14) (Table 7).

At 3-year follow-up, 10 patients (13.2%) had died. The 3-year overall survival (OS) rate for the cohort was 89.5%.

**Figure 4:**
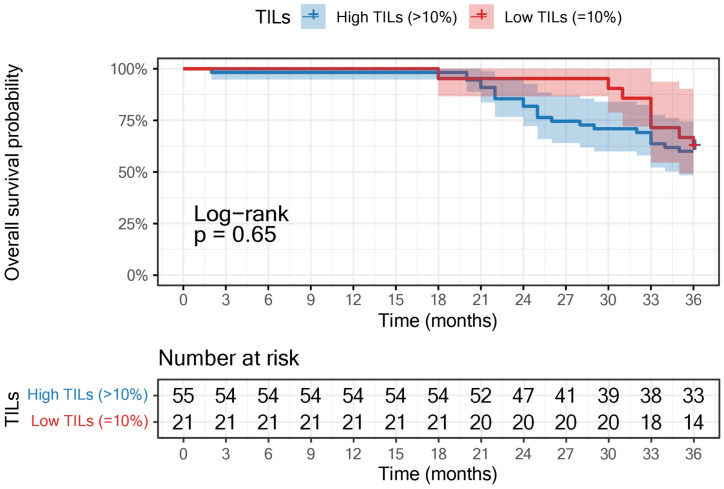
Kaplan–Meier estimate of 3-year overall survival stratified by high (>10%) vs. low (≤10%) TIL infiltration level.

No significant difference in 3-year OS was observed between patients with high (>10%) and low (≤10%) TIL infiltration. The Kaplan–Meier curves overlapped substantially, and the log-rank test did not indicate a statistically significant separation (*p* = 0.65; Figure 4).

**Figure 5:**
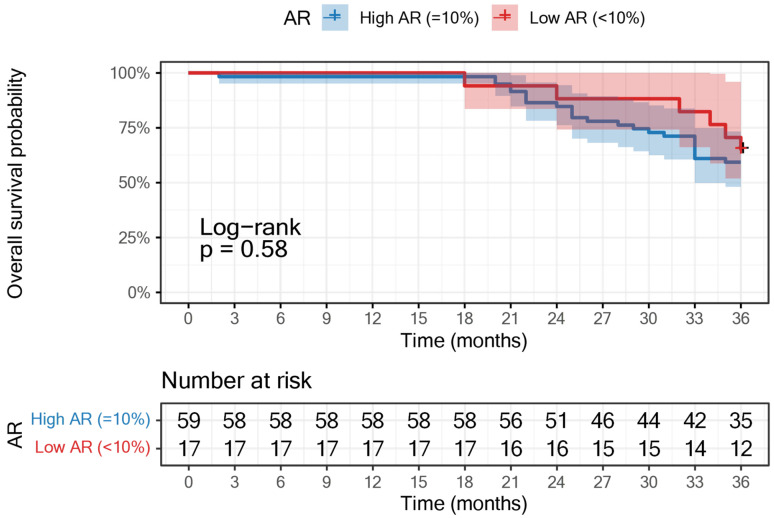
Kaplan–Meier estimate of 3-year overall survival, stratified by high (≥10%) vs. low (<10%) AR expression.

Similarly, AR status was not associated with statistically significant differences in short-term survival; no significant difference in 3-year OS was found between AR-positive and AR-negative tumors (*p* = 0.58; Figure 5).

**Figure 6:**
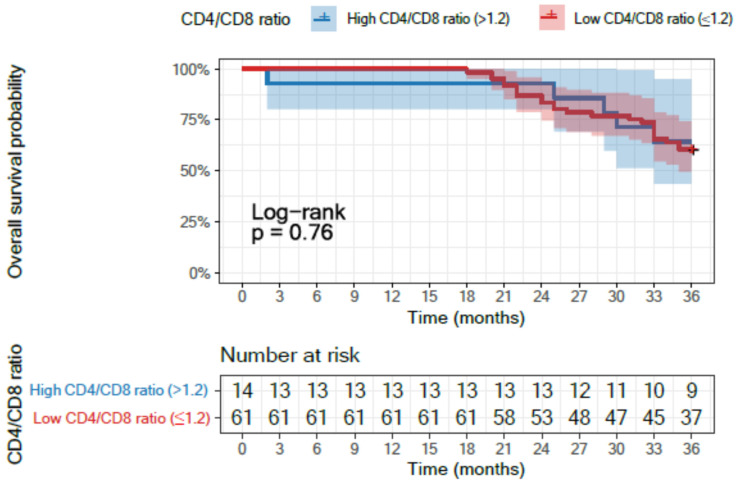
Kaplan–Meier estimate of 3-year overall survival stratified by high (>1.2) vs. low (≤1.2) CD4/CD8 ratio.

The CD4/CD8 ratio was high (>1.2) in 82% of cases. No statistically significant association was observed between CD4/CD8 ratio and 3-year OS (log-rank *p* = 0.76), although a trend was observed in multivariable Cox regression (HR = 1.35, 95% CI: 0.95–1.92, *p* = 0.10). Subgroup analysis suggested that in stage III disease, the combination of high AR, high TILs, and high CD4/CD8 ratio may be associated with better prognosis; however, numbers were small and these findings should be regarded as exploratory (Figure 6).

In the multivariable Cox proportional hazards model restricted to patients with non-metastatic disease (stage I–III) and truncated at 36 months of follow-up, none of the examined clinicopathological variables demonstrated a statistically significant association with 3-year OS. Age at diagnosis did not influence the hazard of death (HR = 1.00; 95% CI: 0.97–1.03; *p* = 0.89). Similarly, ECOG performance status ≥ 1 was not associated with worse survival compared with ECOG 0 (HR = 0.80; 95% CI: 0.36–1.78; *p* = 0.59).

Disease stage (advanced vs. early) showed a non-significant trend toward higher mortality risk (HR = 1.37; 95% CI: 0.64–2.96; *p* = 0.42). Continuous measures of Ki-67 proliferation index, AR expression, and TIL infiltration also did not demonstrate statistically significant associations with survival within the 3-year time window (Ki-67: HR = 1.01 per 1% increase, *p* = 0.47; AR: HR = 1.00 per 1% increase, *p* = 0.79; TILs: HR = 1.00 per 1% increase, *p* = 0.97).

Overall, no variable retained statistically significant independent prognostic value in the adjusted model within this limited follow-up period (Table 8).

No statistically significant difference was found among the four groups presented in Table 9 (log-rank *p* = 0.068), but a significant survival advantage was observed for patients with TILs >10% regardless of AR status (*p* = 0.046).

Detailed three-year mortality rates across the combined TIL, AR, and CD4/CD8 subgroups, stratified by tumor stage, are presented in Supplementary Tables S1–S3.

In exploratory unadjusted comparisons (Supplementary Table S2), higher immune infiltration metrics (sTILs, CD4, and CD8) were statistically significantly associated with 3-year OS. However, these results reflect univariable screening and do not account for clinical covariates, the restriction of survival modeling to stages I–III of disease, or the truncation of follow-up at 36 months. In the prespecified multivariable Cox model restricted to non-metastatic disease, none of the evaluated biomarkers reached statistical significance, which should be interpreted with caution given the limited number of patients and the resulting limited statistical power. Accordingly, we present the unadjusted signals as hypothesis-generating rather than definitive evidence of prognostic value in this cohort.

## 4. Discussion

Our study contributes to the ongoing effort to identify clinically accessible biomarkers that capture the biological heterogeneity of triple-negative breast cancer (TNBC). By integrating androgen receptor (AR) expression with stromal tumor-infiltrating lymphocytes (sTILs) and T-cell subset metrics (CD4, CD8, and CD4/CD8 ratio), we aimed to explore AR–immune relationships using methods feasible in routine pathology. While previous studies has established the prognostic relevance of immune infiltration in TNBC and consensus initiatives have standardized stromal TIL assessment [7,9,10,11], the interaction between AR expression and immune response remains less consistently characterized across cohorts and endpoints [12,13,17].

In our cohort, high AR expression (≥10%) was observed in approximately one-fourth of cases and was strongly associated with lower tumor grade, consistent with the notion that AR-positive TNBC can exhibit a more differentiated phenotype [5,12]. Importantly, we observed that AR-high tumors had significantly lower CD8^+^ infiltration, suggesting a potential AR-associated immune modulation and supporting the concept of biologically distinct AR–immune phenotypes in TNBC. This finding is biologically plausible in the context of emerging subtype frameworks that link AR-enriched/LAR-like TNBC with distinct immune features and genomic profiles [18,19]. Moreover, recent data from a large population-based cohort using objective AR quantification, including digital image analysis, indicate that AR can demonstrate “dual” prognostic behavior—associating with favorable features such as lower grade and lower proliferation while also correlating with unfavorable characteristics such as lymph node positivity and metastatic risk [17]. Together, these observations support interpreting AR not as a universal prognostic marker but as a context-dependent feature whose clinical impact may vary across clinicopathological subgroups.

The role of immune infiltration in TNBC prognosis is well documented, and pooled analyses, consensus statements, and more recent reviews have reinforced the association between higher stromal TILs and improved outcomes in early-stage TNBC [7,8,9,10,11,22]. In our cohort, low TILs were associated with higher grade, consistent with prior observations that immune-depleted tumors often show more aggressive features. However, when focusing on 3-year overall survival (OS) with follow-up truncated at 36 months and multivariable modeling restricted to stage I–III disease, we did not demonstrate statistically significant independent associations between TILs (or CD4/CD8 metrics) and OS. Crucially, this should not be overinterpreted as the absence of prognostic value. The number of events was low (10 deaths at 3 years), limiting statistical power and inflating uncertainty, which can obscure true associations even when effect directions are clinically meaningful.

This point is particularly relevant when reconciling unadjusted and adjusted results. In exploratory univariable screening (Supplementary Table S2), immune metrics (TILs, CD4, CD8) showed statistically significant associations with 3-year OS. In contrast, the prespecified multivariable Cox model restricted to stage I–III disease and truncated at 36 months did not identify statistically significant independent effects. The most parsimonious interpretation is that the unadjusted signals are hypothesis-generating and that adjusted survival inference is constrained by limited event numbers, potential overfitting, and wide confidence intervals.

Our results also underscore that biomarker thresholds remain a practical challenge. International recommendations support standardized TIL assessment and emphasize continuous reporting; nonetheless, categorical cut-offs differ across studies and clinical endpoints [8,9]. For example, neoadjuvant pCR-focused work has used empirically selected thresholds such as ≥40% TILs and highlighted that optimal cut-offs are not universally established [16]. Similarly, AR positivity thresholds vary across studies, contributing to inconsistent prevalence estimates and conflicting prognostic conclusions; recent objective quantification studies support ≥10% as a meaningful cut-off in certain TNBC settings [17]. For the CD4/CD8 ratio, tumor-based thresholds are not standardized in TNBC, and our dichotomization (>1.2), based on prior breast cancer literature, should therefore be regarded as exploratory [21].

From a translational perspective, AR remains a potentially actionable pathway in TNBC. Anti-androgen strategies have been evaluated in clinical studies, including: feasibility data for adjuvant enzalutamide in early-stage AR-positive TNBC and real-world evidence of clinical benefit in a subset of metastatic AR-positive TNBC treated with anti-androgens. Nevertheless, predictive biomarkers remain an unmet need [14,15]. These developments heighten interest in routine, reproducible AR assessment and in understanding AR–immune contexture, particularly as treatment options for biologically selected TNBC subgroups continue to evolve [17,23]. Notably, contemporary cohort studies suggest that AR/TIL phenotyping may identify clinically distinct groups, including AR-positive/TIL-low tumors that are resistant to neoadjuvant chemotherapy [18]. Such findings provide a biologically coherent framework in which our AR–CD8 association may contribute to future stratification strategies.

This study has important limitations. It is retrospective and single-center, and survival inference is constrained by the limited number of patients within the 3-year window, which reduces power for multivariable prognostic modeling. In addition, BRCA status was missing for most patients, limiting evaluation of genetic context. Although outcome blinding was implemented for immune scoring, a formal interobserver reproducibility assessment was not performed; given the documented variability in TIL scoring between observers, this may affect generalizability [20,24]. Finally, biomarker thresholds, particularly for CD4/CD8, are not standardized in TNBC; therefore, subgroup analyses should be interpreted as exploratory.

In summary, our data support the view that AR expression and immune infiltration capture complementary aspects of TNBC biology. High AR expression was linked to reduced CD8^+^ infiltration and lower grade, suggesting an AR-associated immune contexture that may be relevant for future therapeutic stratification. However, the absence of statistically significant independent associations with survival within 3 years should be interpreted with caution due to limited power. Larger prospective cohorts with longer follow-up and standardized biomarker thresholds are needed to validate prognostic utility and to clarify whether AR–immune phenotypes can inform selection of AR-targeted or immunotherapy-based strategies [17,18,25].

## 5. Conclusions

In this retrospective, treatment-naïve TNBC cohort, AR expression was associated with tumor differentiation and immune contexture, most notably with reduced CD8^+^ infiltration in AR-high tumors, supporting the concept of biologically distinct AR–immune phenotypes.

Within a truncated 3-year follow-up window, we did not demonstrate statistically significant independent associations between AR, stromal TILs, or T-cell subset metrics and overall survival in adjusted models. However, these findings should be interpreted with caution, given the limited number of events and the resulting limited statistical power.

Together, our results suggest that AR and immune infiltration reflect complementary aspects of TNBC biology rather than providing definitive short-term prognostic stratification in this cohort. The observed association between AR expression and lower CD8^+^ infiltration may nevertheless be biologically and clinically relevant, particularly in the context of emerging AR-based and immune-oriented therapeutic strategies in TNBC.

Larger prospective cohorts with longer follow-up, standardized biomarker assessment, and validated thresholds are needed to clarify the prognostic utility of AR, TILs, and immune subset metrics and to determine whether AR-associated immune phenotypes may help guide future patient stratification and treatment selection.

## Figures and Tables

**Table 1:** Inclusion criteria.

Description	Criterion
Stage I–IV TNBC (clinical TNM)	Stage
ECOG performance status 0–2	Performance status
Age ≥ 18 years	Age
No prior systemic treatment for breast cancer	Previous treatment
Availability of formalin-fixed paraffin-embedded (FFPE) specimen from the primary tumor	Sample availability

The following clinicopathological data was retrieved: age at diagnosis, menopausal status, family history, BRCA1/2 status, body mass index (BMI), ECOG score, tumor histological type, grade (G), Ki-67 index, and TNM classification according to AJCC (8th edition). BRCA1/2 status was unavailable for most patients (missing in 78%), which limited the ability to evaluate genetic status in association with biomarkers and outcomes, which precluded its inclusion in prognostic modeling.

**Table 2:** Primary antibodies and reagents used in this study.

Manufacturer	Product No.	Dilution	Clone	Target
Dako/Agilent (Glostrup, Denmark)	IR649	RTU	4B12	CD4
Dako/Agilent	IR623	RTU	C8/144B	CD8
Dako/Agilent	M3562	1:25	AR441	AR

All slides were counterstained with hematoxylin and mounted using the Dako Coverslipper.

**Table 3:** Baseline characteristics of the study population.

	Overall
Characteristic	N = 86
Age at diagnosis (years)	
Mean ± SD	55.94 ± 14.01
Median [Q1, Q3]	56.50 [45.00, 66.00]
Min-max	28.00–87.00
Menopausal status, n (%)	
Perimenopausal state	31 (36)
Postmenopausal state	55 (64)
Body mass index (kg/m^2^)	
Mean ± SD	26.70 ± 5.84
Median [Q1, Q3]	24.95 [23.23, 30.83]
Min–max	16.82–42.68
BMI category, n (%)	
(18.5, 25>)	40 (47)
(25, 30>)	20 (23)
>30	22 (26)
≤18.5	4 (4.7)
ECOG performance status, n (%)	
0	52 (60)
1	31 (36)
2	3 (3.5)
Genetic status (BRCA), n (%)	
BRCA1/2−	4 (21)
BRCA1/2+	15 (79)
Missing	67
Ki-67 proliferation index	
Mean ± SD	0.56 ± 0.25
Median [Q1, Q3]	0.60 [0.35, 0.80]
Min-max	0.05–0.95
T category, n (%)	
1	31 (36)
2	35 (41)
3	13 (15)
4	7 (8.1)
N category, n (%)	
0	50 (58)
1	21 (24)
2	11 (13)
3	4 (4.7)
M category, n (%)	
0	76 (88)
1	10 (12)
Anatomical stage (AJCC Anatomic Stage Groups), n (%)	
I	26 (30)
IIA	25 (29)
IIB	9 (10)
IIIA	10 (12)
IIIB	4 (4.7)
IIIC	2 (2.3)
IV	10 (12)

**Table 4:** Characteristics of the study group according to the TILs and AR.

	Overall (N = 86)
Variable	n (%)
AR detected (any expression), n (%)	
No	62 (72)
Yes	24 (28)
AR expression	
Mean ± SD	0.17 ± 0.32
Median [Q1, Q3]	0.00 [0.00, 0.05]
Min-max	0.00–0.95
TILs detected (any infiltration), n (%)	
No	0 (0)
Yes	86 (100)
TIL infiltration	
Mean ± SD	0.28 ± 0.24
Median [Q1, Q3]	0.20 [0.10, 0.40]
Min-max	0.01–0.90
Low AR expression (<10%), n (%)	
Yes	66 (77)
No	20 (23)
High AR expression (≥10%), n (%)	
Yes	20 (23)
No	66 (77)
Low TIL infiltration (≤10%), n (%)	
Yes	28 (33)
No	58 (67)
High TIL infiltration (≥60%), n (%)	
Yes	13 (15)
No	73 (85)
High CD4/CD8 ratio (>1.2), n (%)	
Yes	70 (82)
No	15 (18)
Unknown	1

**Table 5:** Comparison of mean TILs, CD4, CD8, and CD4/CD8 ratio by AR expression level (<10% vs. ≥10%).

	High AR Expression (≥10%)			
Characteristic	No N = 66	Yes N = 20	*p*-Value ^1^	Difference (95% CI) ^2^
TILs			0.16	0.08 (−0.03 to 0.20)
Mean ± SD	0.30 ± 0.24	0.22 ± 0.22		
Median [Q1, Q3]	0.20 [0.10, 0.40]	0.17 [0.06, 0.27]		
Min-max	0.01–0.90	0.05–0.80		
CD4			0.39	0.04 (−0.05 to 0.12)
Mean ± SD	0.19 ± 0.17	0.16 ± 0.16		
Median [Q1, Q3]	0.15 [0.05, 0.30]	0.13 [0.05, 0.20]		
Min-max	0.01–0.70	0.01–0.60		
CD8			0.020	0.04 (0.01 to 0.08)
Mean ± SD	0.10 ± 0.08	0.06 ± 0.06		
Median [Q1, Q3]	0.08 [0.05, 0.15]	0.05 [0.02, 0.05]		
Min-max	0.00–0.30	0.01–0.20		
CD4/CD8 ratio			0.079	−0.63 (−1.3 to 0.08)
Mean ± SD	2.17 ± 1.35	2.80 ± 1.36		
Median [Q1, Q3]	2.00 [1.50, 3.00]	2.75 [1.58, 4.00]		
Min-max	0.33–9.00	0.25–5.00		
Unknown	1	0		

^1^ Permutation Welch Two Sample *t*-test. ^2^ Welch Two Sample t-test. Abbreviation: CI = Confidence Interval.

**Table 6:** Associations between low TIL infiltration (≤10%), tumor grade and Ki-67 proliferation index.

Low TIL Infiltration (≤10%) Characteristic	No N = 58	Yes N = 28	*p*-Value ^1^
G, n (%)			0.039
1	1 (1.7)	1 (3.6)	
2	19 (33)	16 (57)	
3	38 (66)	11 (39)	
High Ki-67 proliferation index (≥20%), n (%)	54 (93)	26 (93)	>0.99

^1^ Fisher’s exact test.

**Table 7:** Associations between high AR expression (≥10%),tumor grade and Ki-67 proliferation index.

	High AR Expression (≥10%)		
Characteristic	No N = 66	Yes N = 20	*p*-Value ^1^
G, n (%)			<0.001
1	0 (0)	2 (10)	
2	18 (27)	17 (85)	
3	48 (73)	1 (5.0)	
High Ki-67 proliferation index (≥20%), n (%)	63 (95)	17 (85)	0.14

^1^ Fischer’s exact test.

**Table 8:** Multivariable Cox proportional hazards model for 3-year overall survival in stage I–III triple-negative breast cancer.

Characteristic	HR (95% CI)	*p*-Value
Age at diagnosis (years)	1.01 (0.98 to 1.04)	0.69
ECOG performance status (≥1 vs. 0)		
0	—	
≥1	0.80 (0.36 to 1.77)	0.58
Disease stage (advanced vs. early)		
Early	—	
Advanced	1.81 (0.79 to 4.14)	0.16
CD4/CD8 ratio	1.35 (0.95 to 1.92)	0.10
Ki-67 proliferation index (% per 1% increase)	1.01 (0.99 to 1.03)	0.35
AR expression (% per 1% increase)	0.99 (0.97 to 1.01)	0.40
TIL infiltration (% per 1% increase)	1.00 (0.98 to 1.02)	0.90

Abbreviations: CI = Confidence Interval, HR = Hazard Ratio.

**Table 9:** Combined TIL/AR subgroups and 3-year mortality.

**Mortality at 3 Years**	**AR Expression**	**TIL Level**	**Group**
15.90%	AR = 0%	>10%	I
31.60%	AR = 0%	≤10%	II
0%	AR > 0%	>10%	III
33.30%	AR > 0%	≤10%	IV

## Data Availability

Data are contained within the article or in the Supplementary Material.

## Supplementary Materials

The following supporting information can be downloaded at https://www.mdpi.com/article/10.3390/biomedicines14061325/s1, Table S1: Characteristics of the study group according to 3-year OS depending on standard assessed parameters; Table S2: Characteristics of the study group according to 3-year OS depending on newly assessed parameters; Table S3: Three-year mortality across combined TILs, AR and CD4/CD8 subgroups stratified by tumor stage.
