# Supplementary material for: Androgen Receptor Expression and T-Lymphocyte Infiltration as Prognostic Indicators in Triple-Negative Breast Cancer: A Retrospective Study
Source: Biomedicines. 2026 Jun 11;14(6):1325. doi: 10.3390/biomedicines14061325 (PMC13297410; doi:10.3390/biomedicines14061325)
Supplement: Supplementary file 1 [file biomedicines-14-01325-s001.zip › biomedicines-4252912-supplementary.pdf]

Supplementary materials

Supplementary Table S1. Characteristics of the study group according to 3-year OS depending on standard assessed parameters.

p-value	Test Statistic	Standard Assessed Parameters
0.047	-1.979	T
<0.001	-3.352	N
<0.001		M
0.857		G
0.753	0.314	Ki-67

Supplemetary Table S2. Characteristics of the study group according to 3-year OS depending on newly assessed parameters.

p-value	Test Statistic	Newly Assessed Parameters
0.009	2.597	TILs
0.008	2.638	CD4
0.024	2.253	CD8
0.532	0.624	AR

Supplemantary Table S3. Three-year mortality across combined TILs, AR and CD4/CD8 subgroups stratified by tumor stage.

Tumor stage	TILs / AR / CD4/CD8 subgroup	N	Deaths at 3-year 3 years mortality	95% CI (Wilson)
Advanced stage (III)	High TILs (>10%) | AR = 0% | High CD4/CD8 ratio (>1.2)	6	4 66.7%	30.0%–90.3%
Advanced stage (III)	Low TILs (≤10%) | AR = 0% | High CD4/CD8 ratio (>1.2)	1	1 100.0%	20.7%–100.0%
Advanced stage (III)	High TILs (>10%) | AR > 0% | High CD4/CD8 ratio (>1.2)	4	1 25.0%	4.6%–69.9%

Tumor stage	TILs / AR / CD4/CD8 subgroup	N	Deaths at 3-year 3 years	mortality	95% CI (Wilson)
Advanced stage (III)	Low TILs (≤10%) | AR > 0% | High CD4/CD8 ratio (>1.2)	1	0	0.0%	0.0%–79.3%
Advanced stage (III)	High TILs (>10%) | AR = 0% | Low CD4/CD8 ratio (≤1.2)	1	1	100.0%	20.7%–100.0%
Advanced stage (III)	Low TILs (≤10%) | AR = 0% | Low CD4/CD8 ratio (≤1.2)	1	1	100.0%	20.7%–100.0%
Advanced stage (III)	High TILs (>10%) | AR > 0% | Low CD4/CD8 ratio (≤1.2)	2	0	0.0%	0.0%–65.8%

Tumor stage	TILs / AR / CD4/CD8 subgroup	N	Deaths at 3-year 3 years mortality	95% CI (Wilson)
Early stage (I-II)	High TILs (>10%) | AR = 0% | High CD4/CD8 ratio (>1.2)	28	10 35.7%	20.7%–54.2%
Early stage (I-II)	Low TILs (≤10%) | AR = 0% | High CD4/CD8 ratio (>1.2)	7	3 42.9%	15.8%–75.0%
Early stage (I-II)	High TILs (>10%) | AR > 0% | High CD4/CD8 ratio (>1.2)	9	4 44.4%	18.9%–73.3%
Early stage (I-II)	Low TILs (≤10%) | AR > 0% | High CD4/CD8 ratio (>1.2)	5	2 40.0%	11.8%–76.9%

Tumor stage	TILs / AR / CD4/CD8 subgroup	N	Deaths at 3-year 3 years mortality	95% CI (Wilson)
Early stage (I-II)	High TILs (>10%) | AR = 0% | Low CD4/CD8 ratio (≤1.2)	5	2 40.0%	11.8%–76.9%
Early stage (I-II)	Low TILs (≤10%) | AR = 0% | Low CD4/CD8 ratio (≤1.2)	5	1 20.0%	3.6%–62.4%
